# 3-Oxoacyl-ACP Reductase from *Schistosoma japonicum*: Integrated *In Silico*-*In Vitro* Strategy for Discovering Antischistosomal Lead Compounds

**DOI:** 10.1371/journal.pone.0064984

**Published:** 2013-06-07

**Authors:** Jian Liu, Dave Dyer, Jipeng Wang, Shuqi Wang, Xiaofeng Du, Bin Xu, Haobing Zhang, Xiaoning Wang, Wei Hu

**Affiliations:** 1 State Key Laboratory of Genetic Engineering, Department of Microbiology and Microbial Engineering, School of Life Sciences, Fudan University, Shanghai, China; 2 Key Laboratory of Parasite and Vector Biology of Ministry of Public Health, Institute of Parasitic Diseases, Chinese Center for Disease Control and Prevention, Shanghai, China; 3 Department of Entomology, University of Wisconsin-Madison, Madison, Wisconsin, United States of America; Florida State University, United States of America

## Abstract

**Background:**

Schistosomiasis is a disease caused by parasitic worms and more than 200 million people are infected worldwide. The emergence of resistance to the most commonly used drug, praziquantel (PZQ), makes the development of novel drugs an urgent task. 3-oxoacyl-ACP reductase (OAR), a key enzyme involved in the fatty acid synthesis pathway, has been identified as a potential drug target against many pathogenic organisms. However, no research on *Schistosoma japonicum* OAR (*Sj*OAR) has been reported. The characterization of the *Sj*OAR protein will provide new strategies for screening antischistosomal drugs that target *Sj*OAR.

**Methodology/Principal Findings:**

After cloning the *Sj*OAR gene, recombinant *Sj*OAR protein was purified and assayed for enzymatic activity. The tertiary structure of *Sj*OAR was obtained by homology modeling and 27 inhibitor candidates were identified from 14,400 compounds through molecular docking based on the structure. All of these compounds were confirmed to be able to bind to the *Sj*OAR protein by BIAcore analysis. Two compounds exhibited strong antischistosomal activity and inhibitory effects on the enzymatic activity of *Sj*OAR. In contrast, these two compounds showed relatively low toxicity towards host cells.

**Conclusions/Significance:**

The work presented here shows the feasibility of isolation of new antischistosomal compounds using a combination of virtual screening and experimental validation. Based on this strategy, we successfully identified 2 compounds that target *Sj*OAR with strong antischistosomal activity but relatively low cytotoxicity to host cells.

## Introduction

Schistosomiasis is a major tropical endemic disease in developing countries. It is estimated that 779 million people in 76 countries and territories are at risk of this disease, which results in approximately 28,000 deaths annually [1], [2]. In China, *S. japonicum* is the major pathogen of this disease [3]. Currently, the treatment and control of schistosomiasis depends almost exclusively on praziquantel (PZQ). This drug has been widely used for nearly 40 years because of its high efficiency and low cost [4]. However, it is speculated that long-term utilization of a single drug could accelerate the emergence of drug-resistant parasites. Decreased sensitivity of *Schistosoma mansoni* and *Schistosoma haematobium* to PZQ has already been reported [5], [6]. Although no reduced sensitivity of *S. japonicum* has been shown to date, the efficacy of this drug varies in different strains of this species [7]. Thus, developing new antischistosomal lead compounds to curb the emergence of drug-resistant schistosomes should be given a high priority.

Fatty acids are prominent and essential components of phospholipids and sphingolipids that constitute the plasma membrane and the membranes of various organelles [8], [9]. Furthermore, they also play important roles in cell signaling and energy storage through the formation of triglycerides [10]. Fatty acid synthesis sequentially goes through repetitive cycles of condensation, β-reduction, dehydration, and enoyl reduction, which are catalyzed by β-ketoacyl synthase, β-ketoacyl reductase, β-hydroxyacyl dehydrase, and enoyl reductase, respectively [11]. Based on the different architecture of the enzymes involved in this pathway, fatty acid synthesis (FAS) can be classified into two types [12]. The FASI system is present in most eukaryotes (except plants) and is characterized by a multidomain polyprotein that encodes all enzymes necessary for fatty acid synthesis in one large polypeptide [13]. By contrast, the FASII system is found in bacteria and parasites, as well as mitochondria and chloroplasts. In the FASII system, each reaction of FAS is catalyzed by discrete enzymes [14]. Some Actinobacteria (e.g. Mycobacteria, Corynobacteria, Nocardia) utilize both the FASI and FASII systems [15].

Unlike most organisms, schistosomes are unable to synthesize fatty acids *de novo*, yet are able to modify fatty acids that they obtain from their host by chain elongation [16]. This unique characteristic inspired us to further investigate the fatty acid elongation pathway in schistosomes. Although schistosomes are eukaryotes, they appear to use a fatty acid production system analogous to some Actinobacteria. Both Actinobacteria and schistosomes not only have a multifunctional fatty acid synthase of the bacterial type, but also some discrete enzymes involved in the FASII system (http://www.kegg.jp/kegg-bin/show/pathway, http://lifecenter.sgst.cn/Schistosoma/cn/pathway) (Figure S1). Despite the individual reaction steps of FASI and FASII being fundamentally similar, the products are different. The FAS-I system is responsible for the biosynthesis of C_16_ and C_18_ fatty acids, the normal products of *de novo* synthesis, while FAS-II further elongates FASI products to generate fatty acids containing longer carbon chains, which usually have specific functions [17]. Meanwhile, given that the enzymes involved in the FASII system generally lack overall sequence homology with the enzymes involved in the mammalian FASI pathway, it is reasonable to consider the schistosome Type II fatty acid synthesis pathway as an attractive pathway for developing new antischistosomal drugs, since its inhibition is unlikely to be harmful to the host [18].

3-oxoacyl-ACP reductase (OAR), also known as β-ketoacyl reductase (KR), is the second enzyme in the Type II fatty acid elongation cycle. It is an NADPH-dependent enzyme that reduces the β-keto group of β-ketoacyl-ACP to β-hydroxyl [19]. OAR belongs to the family of short-chain dehydrogenase/reductases (SDR). Although the sequence similarity between different SDR proteins is not high (usually from 15% to 30%), the crystal structures of all SDR proteins that have been resolved share a highly conserved α/β “sandwich” folding pattern, which represents a typical Rossmann-fold motif [20]. OARs usually exist as a tetramer in solution, and the residues Ser138, Tyr151, and Lys155 (sequence numbers of *Escherichia coli* OAR) are grouped together to formed the catalytic region [19]. Previous studies have demonstrated that OARs are prospective drug targets for control and treatment of tuberculosis, malaria and respiratory infections (caused by *S. pneumoniae*) [11], [21], [22]. These results implied that *Sj*OAR might be also considered as a drug target of schistosomiasis.

In this study, we identified 30 small molecules through molecular docking and then assessed their antischistosomal activity *in vitro*. To further confirm the reliability of the drug target, we investigated the inhibition of the selected active compounds on the activity of recombinant *Sj*OAR (r*Sj*OAR) protein. Finally, the cytotoxicity against host cells was analyzed. Eventually, two compounds were identified that possessed good antischistosomal activity but relatively low cytotoxicity, making them good starting candidates for antischistosomal compounds. Additionally, most of the previous studies focused on either the screening of inhibitors of a known drug target or antischistosomal activity analysis of potential drugs [23]–[25]. In contrast, this study introduced an effective and economic strategy to effectively bridge the gap between virtual screening and experimental validation to develop novel antischistosomal lead compounds.

## Materials and Methods

### Ethical statement

The animal work was approved by the Ethics Committee of the National Institute of Parasitic Diseases, Chinese Center for Disease Control and Prevention in Shanghai, China (Ref No: 20100525-1). Animal care and all procedures involving animals strictly followed the Guidelines for the Care and Use of Laboratory Animals of the Ministry of Science and Technology of People's Republic of China ([2006]398). All efforts were made to minimize suffering of animals.

### Materials

NADPH was purchased from Roche (Switzerland). Acetoacetyl-CoA (AcAcCoA) and PZQ were obtained from Sigma (USA). Small molecules identified by virtual screening came from the Maybridge HitFinder library (USA). Restriction endonucleases *Bam*HI and *Sal*I were from New England Biolabs (USA). Various chromatographic column resins (Ni Sepharose High performance, CM Sepharose Fast Flow and Sephacry S-200 High Resolution) were all purchased from GE Healthcare Life Sciences (USA). RPMI 1640, DMEM and bovine serum (Newborn calf serum and fetal bovine serum) came from Invitrogen (USA). The pET28a vector, *E. coli* DH5α and BL21 (DE3) strains and Hep G2 cells were stored in our laboratory. *S. japonicum* cercariae were provided by the pathogen biology laboratory of the National Institute of Parasitic Diseases, Chinese Center for Diseases Control and Prevention. Specific pathogen-free Kunming female mice (4–6 weeks old) were purchased from the Shanghai Experimental Animal Centre, Chinese Academy of Sciences (China).

### Cloning of *Sj*OAR

The complete open reading frame (ORF) of *S. japonicum* OAR was amplified by PCR from the EST sequence of clone SJL2-003_D08 using forward primer (5′CGGGATCCATGATATCGCTATCAAAAAAGGTG3′) and reverse primer (5′GCGTCGACCTACCGTGGGCACATGATAG3′) containing *Bam*HI and *Sal*I restriction sites, respectively. Next, the target fragment was subcloned into pET28a vectors and a new recombinant plasmid, *Sj*OAR-pET28a, was constructed. The accuracy of the *Sj*OAR-pET28a plasmid was verified by PCR and double-digestion, and DNA sequencing was performed by Invitrogen Corporation (USA). The basic information of the *Sj*OAR protein such as molecular weight (MW) and isoelectric point (pI) were calculated using the ProtParam tool (http://web.expasy.org/protparam/). The signal peptide was analyzed using the SignalP 3.0 server (http://www.cbs.dtu.dk/services/SignalP). *Sj*OAR orthologs were identified by BLAST searches against the NCBI non-redundant protein sequence database (http://www.ncbi.nlm.nih.gov/BIAST) using *Sj*OAR as a query (ACCESSION: AAW26955.1). Multiple sequence alignment was performed using ClustalW software according to the method previously described [26].

### Expression and purification of r*Sj*OAR

Recombinant *Sj*OAR-pET28a plasmid was transformed into *E. coli* BL21 (DE3) cells and cultured in 1L Luria-Bertani (LB) medium plus 50 µg/ml kanamycin. One mM isopropylthio-β-D-galactoside (IPTG) was added into the cell cultures until the OD_600_ reached 0.8–1.0 to induce the expression of *Sj*OAR. The cells were continuously cultured for 6 h and then harvested by centrifugation at 10, 000 g for 30 min at 4°C. Subsequently, the cells were resuspended in lysis buffer (20 mM Tris-HCl, 500 mM NaCl, 1 mM PMSF, pH7.0) and disrupted with a sonicator for 5 min using 2 s pulses at 160 W (SCIENTZ, China). The whole cell lysate was clarified by centrifugation at 10, 000 g for 30 min at 4°C.

The purification of r*Sj*OAR was sequentially performed in three steps (affinity, ion-exchange and gel filtration). All steps were performed at 4°C to minimize precipitation. First, the resulting supernatant was diluted 3-fold and loaded onto a Ni^2+^-NTA column pre-equilibrated with lysis buffer. The column was washed thoroughly with 50 mM imidazole in lysis buffer to remove contaminants, before eluting r*Sj*OAR with lysis buffer containing 250 mM imidazole. Fractions containing r*Sj*OAR were pooled together followed by dialysis overnight against 20 mM Tris-HCl (pH7.0), 50 mM NaCl. Next, the dialyzed r*Sj*OAR sample was centrifuged at 10, 000 g for 30 min at 4°C to remove aggregates and then loaded onto a cation exchange CM column. The target r*Sj*OAR protein was eluted with an increasing linear NaCl gradient to a final concentration of 1 M in 20 mM Tris-HCl buffer, pH7.0. Gel filtration was used for the last purification step, and the sample buffer was changed to 20 mM Tris-HCl (pH7.0), 100 mM NaCl. The r*Sj*OAR expression and protein purity after each purification step was assessed by SDS-PAGE followed by Coomassie brilliant blue staining. The r*Sj*OAR concentration was determined by Bradford Protein Assay Kit (Glory, USA).

### Recombinant *Sj*OAR enzymatic activity and kinetic analyses

The r*Sj*OAR activity analysis was performed in 50 mM sodium phosphate buffer, pH6.8, containing 250 mM NaCl, 0.2 mM NADPH, 0.5 mM AcAcCoA and 0.15 µM–2.40 µM r*Sj*OAR in a final volume of 100 µl. The mixture was incubated for 5 min at 37°C before the reaction was initiated by adding substrate, NADPH or AcAcCoA. The reaction process was monitored by reduction of absorbance at 340 nm, indicating NADPH depletion, using a Model 680 Microplate Reader (Bio-Rad, USA).

The Michaelis constant [K_m_ (app)] values for NADPH and AcAcCoA were determined by varying the concentration of one substrate while maintaining the other substrate at a fixed saturation level. The linear rates obtained over the first 0–2 min were analyzed by the non-linear regression function in OriginPro software. To investigate the enzyme mechanism, a matrix of kinetic data was taken where one substrate was varied at four fixed concentrations of the other substrate. Individual data sets were analyzed using the Michaelis-Menten equation (eq 1) in order to establish whether the double-reciprocal plots were parallel or convergent. The data for intersecting initial velocity patterns were fitted to eq2, describing a steady-state sequential mechanism.



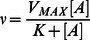
(1)




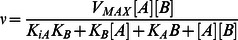
(2)


where ν is the rate, V_MAX_ is the maximal velocity, [A] and [B] are the concentrations of substrates, K_A_ and K_B_ are Michaelis constants for A and B, respectively and K_iA_ is the dissociation constant of substrate A.

### *Sj*OAR homology modeling

To create a 3-Dimensional model of the *Sj*OAR protein, the amino acid sequence was threaded using the template-based structure prediction server HHpred followed by model building with Modeller (Bioinformatics Toolkit-Max-Planck Institute for Developmental Biology). The HHpred prediction server is considered one of the top servers out of the 81 that participated in CASP9 (The Critical Assessment of protein Structure Prediction, version 9) [27]. The template that ranked the highest was levodione reductase from *Corynebacterium aquaticum* M-13 (PDB ID: 1IY8), which is a short-chain dehydrogenase (SDR) bound with its cofactor, NAD and inhibitor, 2-methyl-2, 4-pentanediol (MRD) [28].

### Molecular docking

The Maybridge HitFinder library of 14,400 small molecules was chosen as an ideal docking library that we could first test *in silico* against the model of *Sj*OAR and at a later date *in vitro* against the recombinant protein. The small molecules tested follow the Lipinski guidelines for "drug-likeness" and have properties that include: no more than 5 hydrogen bond donors, no more than 10 hydrogen acceptors, a molecular mass less than 500 Daltons, and an octanol-water partition coefficient log P less than 5 [29].

Molecular docking was conducted by first running the small molecules through the Sybyl v8.0 Surflex-Dock program (www.tripos.com) followed by taking the top 5% of hits and running those through AutoDock 4.2 (http://autodock.scripps.edu/). Prior to docking in Sybyl v8.0, hydrogen atoms were added to the *Sj*OAR model, Gasteiger-Huckel charges were added, and the model was energy minimized. Once the Maybridge HitFinder library had finished docking, the data were exported to an Excel spreadsheet and sorted. The top 5% of hits were then run through AutoDock 4.2, which is more comprehensive yet more CPU-time consuming. To further sort the small molecules, they were examined with Stereo goggles to determine whether improvements could be made to the docking experiment if certain sidechains were allowed to have more flexibility. Post-observation, we decided to generate a new list from the top 5% of targets selected from Sybyl to be run again through AutoDock 4.2, this time allowing the active site residues Ser150 and Tyr163 to exhibit conformational flexibility.

### BIAcore assay

The binding capacities of small compounds identified by the virtual screen with r*Sj*OAR were assessed using a BIAcore 3000 instrument (BIAcore AB, Uppsala, Sweden). The recombinant His-tag fusion protein *Sj*OAR was immobilized onto an NTA chip. Compounds were injected at a concentration of 10 µM and a flow rate of 40 µl/min in HBS buffer (0.01 M HEPES, 0.15 M NaCl, 3.0 mM EDTA, 0.005% Tween 20, pH 7.4) containing 1‰ DMSO (Dimethyl sulfoxide). Electrical alterations on the sensor chip were monitored as a resonance signal by using the program supplied by the manufacturer. To further obtain kinetic parameters, a series of compound concentrations (0.5 µM, 1 µM, 2 µM, 5 µM and 10 µM) were measured. Data were analyzed by using the software with which the instrument was equipped.

### Inhibition studies on cultured worms

Mice infected with cercariae (400–600 cercariae per mice for juvenile worms and 80–100 cercariae per mice for adult worms) were scarified at 14 days and 35 days post-infection for juvenile worms and adult worms, respectively. The freshly perfused worms were washed with saline and then cultured in RPMI 1640 medium supplemented with 300 µg/ml penicillin, 300 µg/ml streptomycin, 0.25 µg/ml amphotericin and 10% fetal bovine serum. Four of adult worms with good viability were transferred into each well of a 24-well Falcon plate containing 2 ml of the above culture medium. Compounds dissolved in DMSO were added to different final concentrations (for initial screening three concentrations 5 µg/ml, 25 µg/ml and 50 µg/ml were evaluated while for the second screening, five concentrations 5 µg/ml, 10 µg/ml, 20 µg/ml, 40 µg/ml and 80 µg/ml were used). Control worms were treated with an equal volume of carrier DMSO or RPMI 1640 medium, and worms treated with PZQ were also evaluated as a positive control. For each experimental condition, 12 worms evenly distributed in 3 wells were tested.

The worms were cultured at 37°C in an incubator with 5% CO_2_. Worm viability, morphological changes and mortality were monitored under an inverted microscope at 2 h, 16 h, 24 h, 48 h and 72 h post-treatment. Parasite death was defined as non-detectable activity for 2 minutes plus morphological and tegumental alterations [30]. Worms with typical lesions were photographed and stored in 2.5% glutaraldehyde in PBS for electron microscope analysis. The median lethal dose (LD_50_) for each active compound was calculated by SPSS 18.0 software, with a confidence interval of 95%. The inhibition study was initially performed with adult worms, and then the compounds identified as active were also tested on juvenile worms with the similar methods described above.

### Effect of active compounds on r*Sj*OAR activity

The effect of antischistosomal compounds on r*Sj*OAR enzymatic activity was measured in the enzyme assays described above. The Median inhibitory concentration (IC_50_) was calculated by curve fitting using SPSS18.0 statistical software with a confidence interval of 95%. All assays were carried out in triplicate and repeated three times.

### Cytotoxicity assay

Cytotoxicity assays were performed with the human liver carcinoma cell line Hep G2 using Cell Counting Kit-8 according to the protocol provided by the supplier (Beyotime, China). Briefly, the cells were cultured in 96-well plates containing 100 µl of DMEM medium (including 10% fetal bovine serum) at a density of 5,000 cells per well at 37°C in 5% CO_2_. The cells were allowed to grow for 24 h and were then treated with different concentrations of compounds (or compound carriers alone). Once cells in the negative control group (DMSO alone) covered more than 90% of the surface of the well, 10 µl of WST-8 chromogenic agent was added to each well, and incubated for 30 min. The plates were read at A_450 nm_ using a Model 680 Microplate Reader (Bio-Rad, USA).

### Scanning electron microscopy

Schistosome samples were fixed in 2.5% glutaraldehyde in PBS at 4°C for 2 h and then washed with PBS three times. The samples were further fixed in 1% Osmium tetroxide buffer. After ethanol dehydration and critical point drying, samples were mounted on microscope stubs, followed by gold sputtering for 3 min in IB-3 ion-sputtering instrument (Hitachi, Japan). A S-520 SEM (Hitachi, Japan) was utilized with an accelerating voltage of 30 kV for the observation.

## Results

### Cloning and Characterization of *Sj*OAR gene

The full cDNA sequence encoding *Sj*OAR was amplified by PCR and successfully subcloned into the pET28a vector. The cDNA of *Sj*OAR contained an ORF of 798 bp, encoding a protein of 266 amino acids with a predicted molecular weight of 28.7 KDa and a calculated isoelectric point of 7.6. Multiple sequence alignment analysis revealed that *Sj*OAR was 87%, 32% and 25% identical to the homologs from *S. mansoni*, *Plasmodium falciparum* and *M. tuberculosis*, respectively. No homologous proteins were identified in *Homo sapiens,* as expected. The predicted active sites obtained by alignment were Ser150, Tyr163 and Lys167 (Figure S2). Further analysis showed the protein sequence did not contain a signal peptide or transmembrane topology (Table 1).

**Table 1:** Comparison of *Sj*OAR with homologous proteins from other species.

Property	Unit	*S. japonicum*	*P. falciparum*	*E. coli*	*M. tuberculosis*
Molecular Weight	KDa	28.7	33.32	22.56	25.56
pI	-	7.61	9.33	6.76	8.76
Signal peptide	-	No	Yes	No	No
Oligmeric state	-	N.D.	Tetramer	Tetramer	Tetramer
K_m_(app)NADPH	μM	93.6±4.2	47.5±4.5	21	26±3
K_m_(app)AcAcCoA	mM	0.55±0.03	0.51±0.05	2.5	0.17±0.02

Data for *S. japonicum* are from the present study; data for *P. falciparum* are from [12], [22]; data for *E. coli* are from [32] and data for *M. tuberculosis* are from [17], [20]. N.D. not determined.

### Expression, purification and identification of r*Sj*OAR

**Figure 1:**
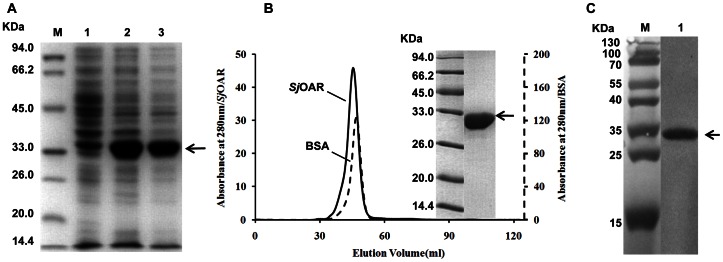
Expression, purification and identification of r*Sj*OAR protein. **A.** SDS-PAGE analysis of *Sj*OAR expression from *E. coli* BL21 transformed with the recombinant plasmid *Sj*OAR-pET28a. M, molecular weight marker; lane 1, whole lysate of bacterial without induction; lane 2 and lane 3, whole lysate and supernatant of bacteria induced with 1 mM IPTG at 37°C for 6 h. **B.** Elution profile of *Sj*OAR on a gel chromatography column. BSA with a molecular weight of 67 KDa (monomer) was used as an internal control. The insert figure shows a single band on an SDS-PAGE gel. **C.** Identification of r*Sj*OAR by western blotting. M, prestained protein marker; lane 1, the purified His-tag fusion *Sj*OAR protein was probed with rabbit anti-His antibody (1:2,000 diluted in PBST buffer). The secondary antibody was horseradish peroxidase (HRP)-conjugated goat anti-rabbit IgG antibody (1: 8,000 diluted in PBST buffer). Chromogenic detection was performed using 3, 3′-diaminobenzidine (Sigma, USA). The arrow indicates the target band, assumed to be *Sj*OAR protein.

The recombinant His-tag fusion *Sj*OAR protein was successfully expressed as soluble protein in the *E. coli* BL21 (DE3) strain, and had a predicted molecular weight of 32.25 KDa (including the N- terminal His-tag) (Figure 1A). After the three-step purification, the resulting *Sj*OAR protein presented as a single band on SDS-PAGE gel, with a single symmetrical peak upon gel filtration (Figure 1B), suggesting that *Sj*OAR is of high-purity and uniform conformation. Additionally, western blotting analysis also indicated the recombinant His-Tag fusion *Sj*OAR protein could be recognized by rabbit anti-His-tag antibody (Figure 1C).

### Activity and kinetics of r*Sj*OAR

**Figure 2:**
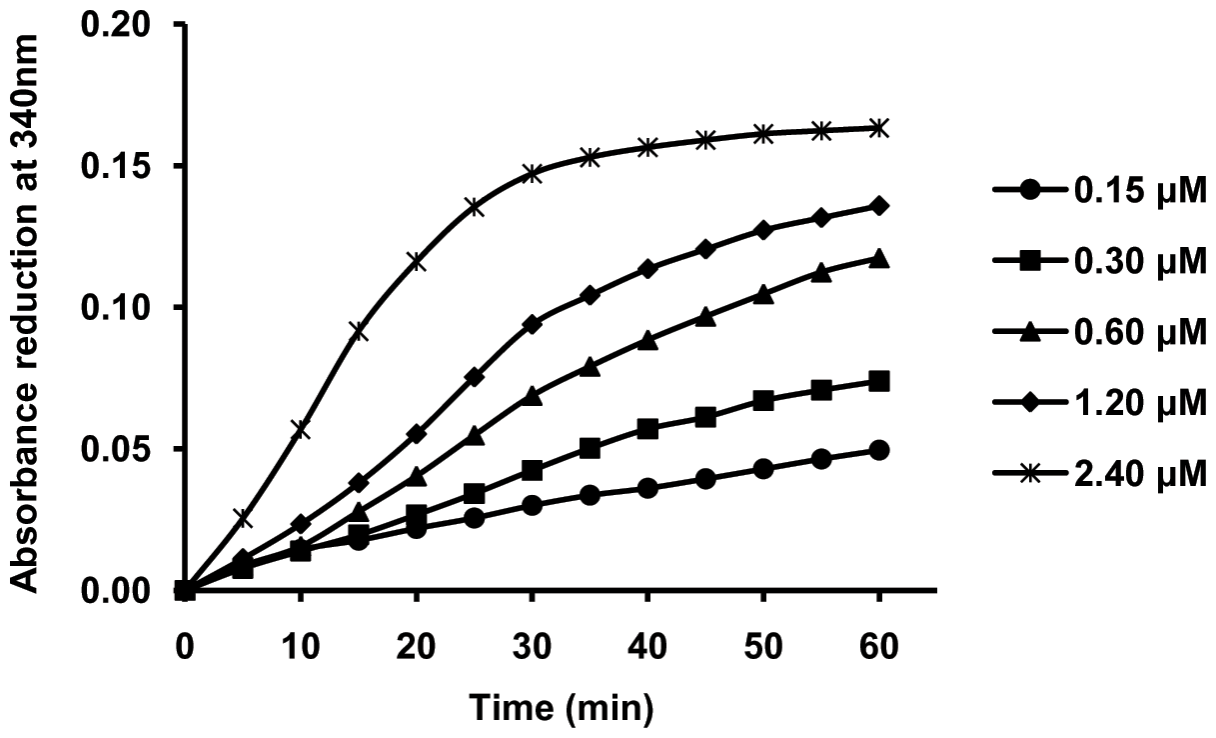
Enzymatic activity of r*Sj*OAR *in vitro*. The concentrations of *Sj*OAR for each reaction were 0.15 µM (circle), 0.30 µM (square), 0.60 µM (triangle), 1.20 µM (diamond) and 2.40 µM (asterisk). The analysis was done in triplicate and repeated three times.

**Figure 3:**
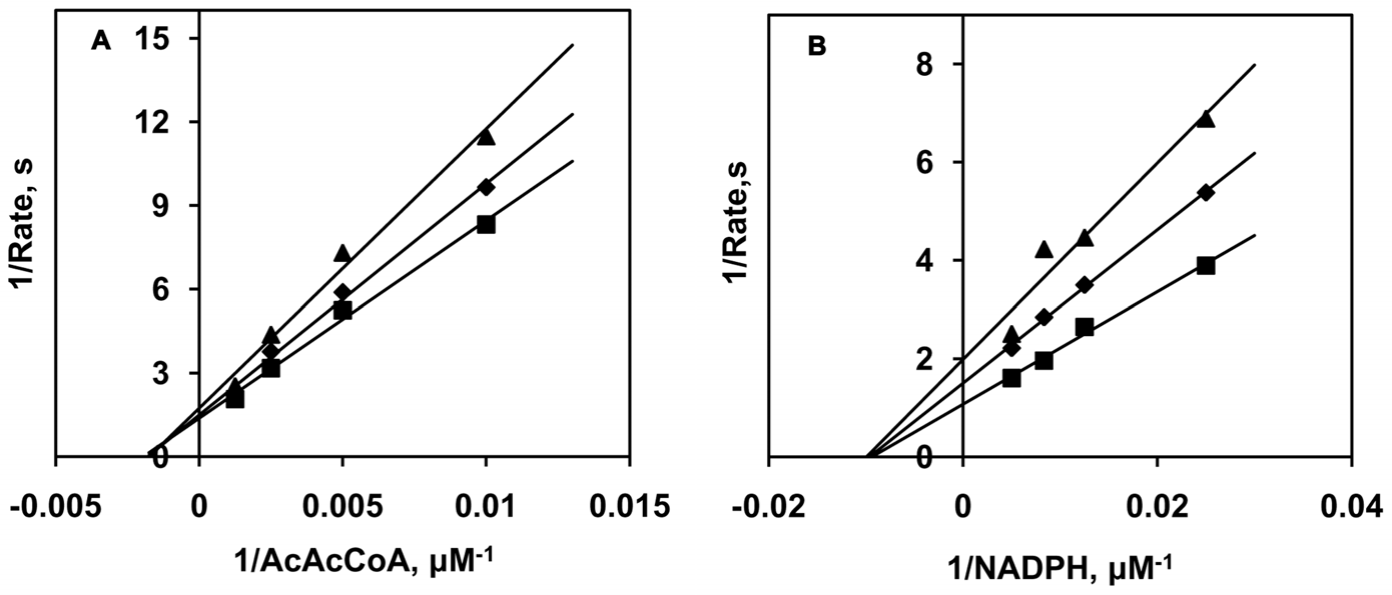
Kinetic analysis of r*Sj*OAR. **A.** AcAcCoA concentration was varied with fixed concentrations of NADPH (triangle, 100 µM; diamond, 150 µM; square, 200 µM). **B.** NADPH concentration was varied with fixed concentrations of AcAcCoA (triangle, 500 µM; diamond 750 µM; square, 1, 000 µM). The rates for each reaction were fitted individually to the Michaelis-Menten equation by non-liner regression and displayed as double-reciprocal plots. The molar absorptivity (ε) of NADPH at 340 nm is 6.22 mM^−1^cm^−1^ [33].

OAR is an NADPH-dependent oxidoreductase that catalyzes 3-oxoacyl-[Acyl-carrier-protein] to 3-oxoacyl-[Acyl-carrier-protein] [31]. Herein, the structural analogue AcAcCoA instead of 3-oxoacyl- [Acyl-carrier-protein] was used to determine the activity of r*Sj*OAR. As shown in Figure 2, a dose-dependent absorbance reduction was observed at 340 nm as a result of NADPH depletion, which indicates that r*Sj*OAR had a measurable activity *in vitro*. To distinguish between a sequential and a ping-pong mechanism, initial reaction rates were determined in the first 2 min, using either AcAcCoA or NADPH as the substrate. Analysis of the double-reciprocal plots revealed intersecting patterns for both substrates (Figure 3), which is consistent with ternary complex formation and a sequential mechanism. Since the intersection was located on the negative side of the x axis, we concluded that the K_m_ values are equal to the apparent K_m_. Similar intersecting initial velocity patterns have been reported for homologous *Sj*OAR proteins from other species (*M. tuberculosis, S. pneumoniae* and *P. falciparum*) [17], [21], [22]. Fitting the data with eq2 yielded the kinetic values of K_m_ (NADPH)  =  (93.6±4.2) µM and K_m_ (AcAcCoA)  =  (0.55±0.03) mM. These values are comparable with other homologous OAR proteins summarized in Table 1.

### Structure-based virtual screening

The predicted overall structure of *Sj*OAR shows a typical α/β “sandwich” folding pattern, with a central β-sheet that is flanked by several α-helices, characteristic of the Rossmann-fold motif. (Figure S3A). The structural conservation of the NAD binding pocket and active sites where the inhibitor MRD resided in the structure of *C. aquaticum* M-13 levodione reductase allowed us to identify an ideal target area to begin docking (Figure S3B). Molecular docking revealed 30 candidate compounds, and their binding free energy with *Sj*OAR are listed in Table 2.

**Table 2:** The top 30 hits identified by molecular docking.

Chemical Registry	Order Number	Renamed	Score	Molecular weight
255725-51-0*	BTB 13500	-	−14.54	382.38
330196-53-7	RJC02308	OAR1	−13.74	419.40
195195-07-4*	DP 00993	-	−13.46	424.55
538337-90-5	HTS10359	OAR2	−13.41	373.43
683205-50-7	HTS01173	OAR3	−13.21	419.52
392718-73-9	HTS06640	OAR4	−13.00	386.48
661451-42-9	CD05160	OAR5	−12.80	370.40
685505-92-4	HTS02028	OAR6	−12.77	412.50
282715-97-3	RB00386	OAR7	−12.73	436.55
26844-12-2	RH01633	OAR8	−12.65	347.46
261623-67-0	SEW06593	OAR9	−12.58	437.60
188791-06-2	HTS00495	OAR10	−12.49	412.42
656221-85-1	CD02517	OAR11	−12.45	395.50
690957-23-4	HTS04083	OAR12	−12.45	393.53
883033-30-5	SPB07931	OAR13	−12.45	412.49
883944-78-3	SCR00964	OAR14	−12.44	345.36
258264-49-2	BTBG00099	OAR15	−12.38	436.54
351857-72-2	RJF01576	OAR16	−12.38	414.84
2167-10-4	PD00329	OAR17	−12.37	377.52
883103-87-5	SCR00087	OAR18	−12.24	388.43
355396-63-3	HTS06142	OAR19	−12.21	349.43
200812-98-2	HTS07411	OAR20	−12.15	419.48
565191-72-2	HTS03705	OAR21	−12.10	370.36
957947-59-0	SCR00783	OAR22	−12.10	423.95
634190-28-6	KM10460	OAR23	−12.06	389.47
883944-84-1*	SCR00968	-	−12.06	426.46
883027-53-0	SP00610	OAR24	−12.04	469.49
683206-13-5	HTS01294	OAR25	−12.02	413.49
287917-46-8	RF03445	OAR26	−12.02	395.91
883054-36-2	XBX00329	OAR27	−11.40	313.65

Details about these compounds can be obtained from http://zinc.docking.org/. Compounds marked with an asterisk were not obtained because of a shortage.

### BIAcore assays

The binding capacity between the identified small molecules and r*Sj*OAR was confirmed by BIAcore analysis. The results showed that a response signal was detected for all identified compounds, while only a small signal was observed with buffer only or with PZQ (Figure S4). The kinetic parameters K_A_ (association constant) and K_D_ (dissociation constant) were obtained by fitting the response values under five different concentrations to the appropriate kinetic model. Kinetic parameters for certain compounds are listed in Table S1. The K_D_ values for most compounds were lower than 50 µM, and some of them reached as low as 10 µM. The highest binding capacity among all the compounds tested was OAR27, with a K_D_ value of 1.85×10^−8^ M (Table S1).

### Inhibition studies on cultured adult worms

**Figure 4:**
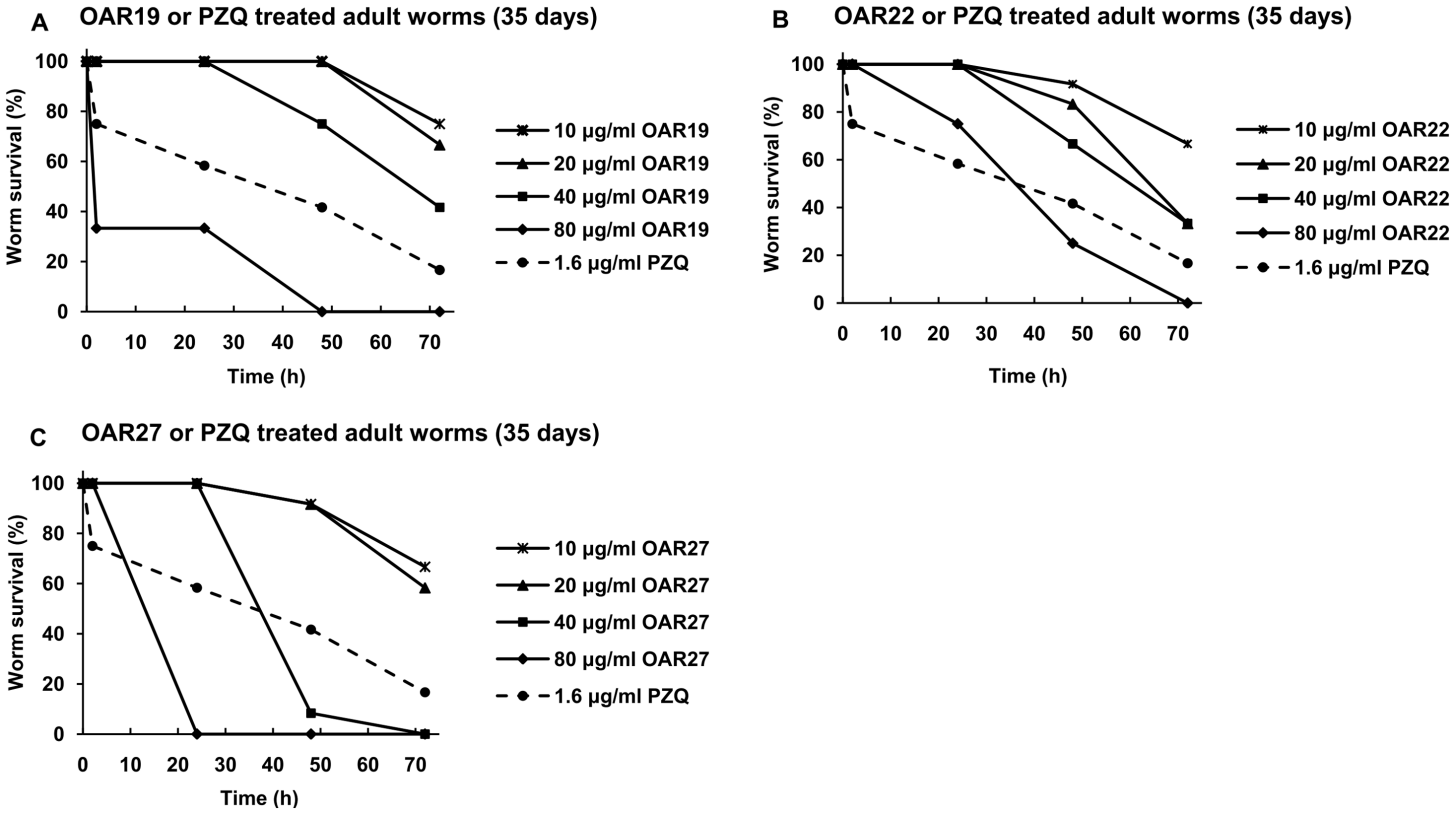
Time-dependent survival rates of adult worms treated with compounds 19, 22, 27 and PZQ. **A**, **B** and **C**, Adult worms were treated with compounds 19, 22 and 27. Compound concentrations were 10 µg/ml (asterisk), 20 µg/ml (triangle), 40 µg/ml (square) and 80 µg/ml (diamond). 1.6 µg/ml PZQ (dash line with circle mark) was used as a positive control.

**Figure 5:**
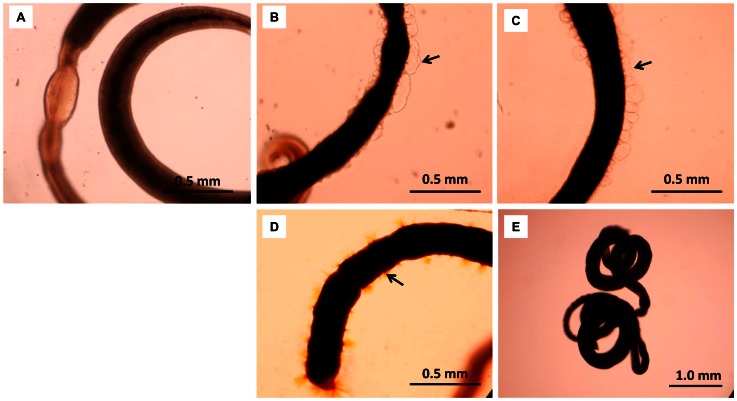
Optical microscopy of worms exposed to compounds 19, 22 and 27. **A.** The morphology of worms treated with 2% DMSO for 72 h. **B.** The morphology of worms treated with 10 µg/ml of OAR19 for 48 h. **C.** The morphology of worms treated with 10 µg/ml of OAR22 for 48 h. **D.** The morphology of worms treated with 40 µg/ml of OAR27 for 24 h. **E.** The morphology of worms treated with 10 µg/ml of PZQ for 24 h.

In the primary screening, five compounds (8, 9, 11, 15 and 24) were identified as having good activity that resulted in 100% mortality after 48 h, but the effects were limited to a concentration of 50 µg/ml. Three compounds (19, 22 and 27) were more effective, resulting in significant mortality rate at a concentration of only 25 µg/ml. These compounds were selected for the secondary test. The effects of compounds 1, 2, 17, 20, 25, 26 were not determined because of their low solubility in DMSO or RPMI 1640 medium, while the rest of the compounds did not exhibit significant activity. To confirm the antischistosomal effects induced by compounds 19, 22 and 27, and further determine their median lethal dose (LD_50_), a secondary screening was performed. At 72 hours, the LD_50_ values of compounds 19, 22 and 27 were (19.7±8.7) µg/ml, (18.0±7.6) µg/ml and (18.5±6.1) µg/ml [corresponding to (56.4±24.9) µM, (42.5±17.9) µM and (59.0±19.4) µM], respectively. Additionally, at higher concentrations (40 µg/ml or 80 µg/ml), OAR19 and OAR27 displayed significant activity in a relatively short time (2 h or 24 h), whereas the antischistosomal activity of OAR22 was slower (Figure 4). The structures of compounds 19, 22, 27 and PZQ are shown in Figure S5. Optical images showed that extensive blebs were observed when worms were treated with 10 µg/ml OAR19 or OAR22 for 48 h (Figure 5 B and 5C). In worms exposed to 40 µg/ml OAR27 for 24 h, lots of thorns were distributed on the tegument (Figure 5D). PZQ induced drastic contractions (Figure 5E), and even at a very low concentration, 1.6 µg/ml (5.1 µM), which resulted in 83.3% mortality in 72 h (Figure 4). Meanwhile, the alterations described above were not observed in the carrier alone (2% DMSO) treated group (Figure 5A).

**Figure 6:**
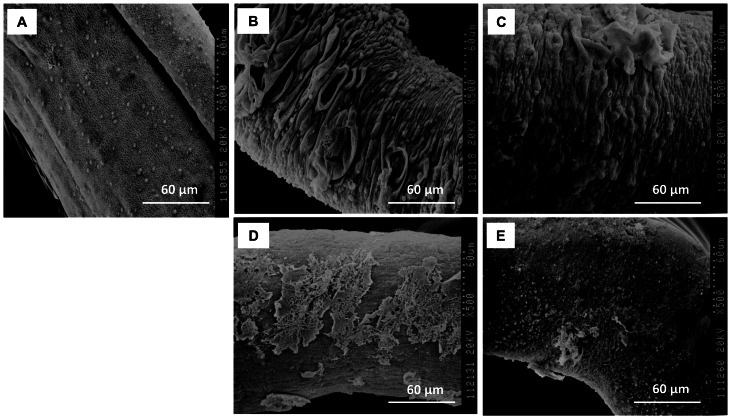
SEM images of the mid-body of adult worms. **A.** The morphology of worms treated with 2% DMSO for 72 h. **B.** The morphology of worms treated with 10 µg/ml of OAR19 for 48 h; **C.** The morphology of worms treated with 10 µg/ml of OAR22 for 48 h. **D.** The morphology of worms treated with 40 µg/ml of OAR27 for 24 h; **E.** The morphology of worms treated with 10 µg/ml of PZQ for 24 h.

Scanning electron microscopy (SEM) images further confirmed the results of the optical microscopy. For the OAR19 and OAR22 treated worms, blebs on the tegument were so severe that they fused together, inducing the most destructive damage on the tegument layer (Figure 6B and 6C). Serious tegument peeling was observed in OAR27 treated worms (Figure 6D). By comparison, the tegument of control schistosomes appeared very smooth, and exhibited a dense network structure (Figure 6A).

### Effect of active compounds on r*Sj*OAR activity

**Figure 7:**
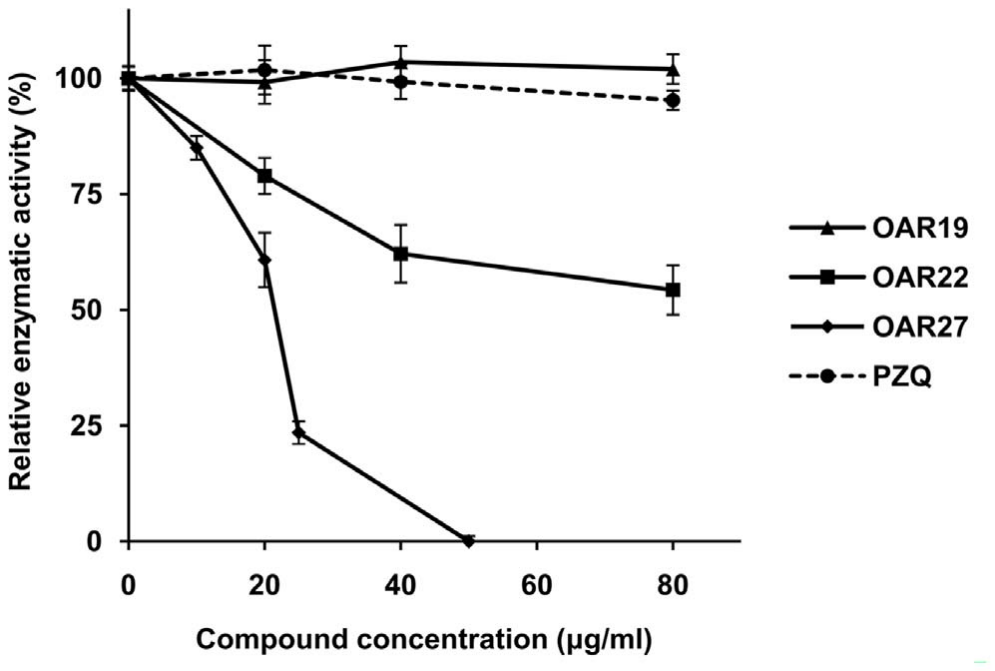
The effect of active compounds on r*Sj*OAR enzymatic activity. OAR19 (triangle), OAR22 (square), OAR27 (diamond) and PZQ (dash line with circle mark). The r*Sj*OAR concentration in each assay was 0.06 µM. Enzymatic activity in the no-inhibitor group was defined as 100%. The inhibition of r*Sj*OAR enzymatic activity in the experimental group was calculated using the following equation: inhibition % = (1−V_i_/V_0_)×100%, where V_0_ is the initial velocity of the no-inhibitor group and V_i_ is the initial velocity in each experimental group.

To further verify whether *Sj*OAR was indeed the drug target of compounds 19, 22 and 27, we assessed their effect on the inhibition r*Sj*OAR enzymatic activity. Compound OAR27 at 25 µg/ml reduced r*Sj*OAR activity by 73% compared with the carrier-treated group. The IC_50_ value was (21.79±1.33) µg/ml [(51.4±3.1) µM] in the first 15 min. In contrast, OAR22 exhibited a poor inhibition effect, and only reduced r*Sj*OAR activity by 45.71%, even at 80 µg/ml. Additionally, OAR19 had no effect on r*Sj*OAR activity, which suggested that OAR19, while exhibiting good antischistosomal activity, was not targeting the *Sj*OAR protein. The commonly used drug PZQ also showed no significant inhibition of r*Sj*OAR activity (Figure 7).

### Cytotoxicity assay

**Figure 8:**
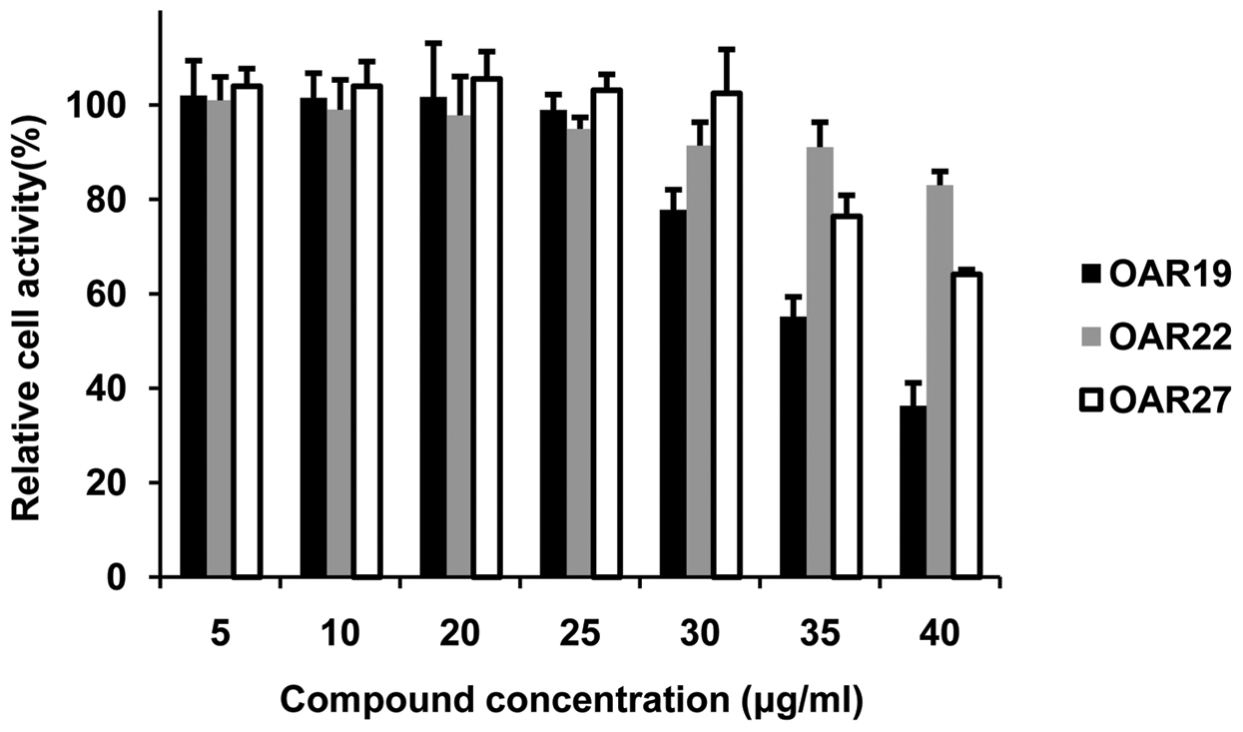
Cytotoxicity assay for human liver cells cultured with different concentrations of active compounds. Columns filled with black, gray and white represent OAR19, OAR22 and OAR27, respectively. The cell activity of the compound-carrier-only group was defined as 100%. The relative cell activity values of other experimental groups were calculated by comparing them with the control group. Data shown in the figure represent three independent experiments.

To assess the cytotoxicity of compounds 19, 22 and 27, the liver carcinoma cell line Hep G2 from *H. sapiens* was used. All compounds exhibited no significant cytotoxicity at their corresponding median lethal dose in worms (approximately 20 µg/ml), but as the concentration increased, the curves exhibited different tendencies. OAR19 cytotoxicity showed a high correlation with concentration, and only about 35% of cells survived at 40 µg/ml. By contrast, the cytotoxicity of OAR22 and OAR27 was mild; 40 µg/ml of OAR22 and OAR27 resulted in 75% and 100% adult worm mortality, while only exhibiting 17% and 36% cytotoxicity in Hep2 cells, respectively (Figure 8).

## Discussion

Fatty acid biosynthesis is essential for the viability of organisms. The associated enzymes comprise exploitable drug-discovery targets, especially in view of the intrinsic differences between mammals (FASI) and prokaryotes (FASII). Schistosomes, although eukaryotes, appear to have both FASI and FASII systems. Many separate enzymes (β-hydroxyacyl dehydrase, FabZ; enoyl reductase, FabK, FabL; and β-ketoacyl reductase, OAR, also named FabG, which was identified in this study) involved in the FASII system have been identified in schistosomes (Figure S1). However, we are not proposing that schistosomes evolved a pathway that similar to Mycobacterium (an organism which had been demonstrated has both FASI and FASII systems); conversely, schistosomes might have retained the prokaryotic FAS II system in the process of evolution. The FASII system exists in many parasitic organisms. As one example, the protozoa *P. falciparum* only employs the FAS II system. Since schistosomes and *P. falciparum* are both blood borne parasitic organisms, they share much in common in certain important metabolic pathways [34]. Therefore, it is reasonable that schistosomes might retain the original FAS-II system, even though it has evolved in other eukaryotes. Additionally, a high content of eicosaenoic acid (C_20_), which is rarely found in mammals, was identified in schistosomal tegumental membranes [35]. As described above, fatty acids with long carbon chains are unlikely to be products of the FASI system, although further study is needed to determine whether they are associated with the FAS II system.

OAR is the second enzyme involved in the FASII system. We performed a BLSATP to search for *Sj*OAR homologues and found OAR homologues exist in flukes (*S. japonicum*, AAW26955.1; *S. mansoni*, XP_002575617.1; *Clonorchis sinensis*, GAA51714.1), Filarial (*Wuchereria bancrofti*, EJW80421.1; *Brugia malayi*, XP_001893058.1), most bacteria, and some marine fish; however, we couldn't identify any *Sj*OAR homologue in mammals, which is consistent with previous studies that concluded the FASII system is absent in mammals. Multiple sequence alignment revealed that *Sj*OAR shares a low sequence identity with homologous proteins from *P. falciparum*, *M. tuberculosis* and *E. coli* (32%, 25% and 30%, respectively), yet possesses conserved active site residues (Ser150, Tyr163 and Lys167), which implies that it might share general catalytic activity with the OARs superfamily (Figure S2). Our enzymatic analysis verified that r*Sj*OAR had measurable activity *in vitro*, and further kinetic analysis showed that the double-reciprocal plot intersected for both substrates, suggesting that the reaction catalyzed by *Sj*OAR follows a steady-state sequential kinetic mechanism (Figure 3). Similar kinetic mechanisms were also reported in *M. tuberculosis* OAR, *P. falciparum* OAR, *S. pneumoniae* OAR and *B. napus* OAR [17], [21], [22], [36].

Homology modeling is a convenient approach to produce protein structural models, especially when the target and templates are closely related. In this study, the *Sj*OAR structure obtained by homology modeling displays a typical α/β folding “sandwich” pattern, with a central β-sheet flanked by several helices (Figure S3), which is similar to previously solved OAR structures from other species [22], [37], [38]. However, it is noteworthy that the predicted structure is just a tentative model, and a more accurate *Sj*OAR structure can only be obtained by experimental methods. We also tried to solve the *Sj*OAR structure using X-ray diffraction, and successfully screened 2 conditions for protein crystallization (unpublished data). Crystal structure optimization is underway. More specific and potent inhibitors could be identified based on the crystal structure of *Sj*OAR. However, *Sj*OAR was found to be prone to aggregate at high protein concentrations, which makes it especially difficult to crystallize.

Using the predicted structural model, we identified two compounds that target *Sj*OAR and show good antischistosomal activity with relatively low cytotoxicity. However, we acknowledge that the compound concentration required to kill adult worms is high in comparison with PZQ. Since juvenile worms are insensitive to PZQ [30], it is valuable to identify whether these compounds can exhibit similar effects on both juvenile and adult worms. Therefore, we tested the effects of OAR22 and OAR27 on juvenile worms (14 days post infection). As shown in Figure S6, 40 µg of OAR22 and OAR27 resulted in 100% mortality in 72 h and 48 h, respectively. By contrast, 40 µg of PZQ only caused 13.5% mortality in the whole experimental period. It is of note that the motor activity of juvenile worms exposed to PZQ decreased significantly in a short time; however, the oral sucker and ventral sucker remained active for a relatively long period, which suggested these worms were still alive. Morphology observation showed that varying degrees of blebs would emerge on the tegumental surface of juvenile worms when they were treated with different concentrations of OAR22. Initially, the blebs were small, becoming larger and more numerous with time. Finally, the enlarged blebs collapsed, and the worms died. The juvenile worms exposed to OAR27 became short and black, and tegument peeling was also observed, which is consistent with the phenomenon seen in adult worms (Figure S7). These results imply that compounds OAR22 and OAR27 exhibit no difference in antischistosomal activity for juvenile and adult worms. This result suggested that these compounds can be used as ancillary compounds with PZQ. We also showed that that PZQ exhibits no inhibition of *Sj*OAR enzymatic activity (Figure 7), which means that drugs targeting *Sj*OAR might have a different mechanism of action. Combination therapies utilizing PZQ and our compounds may be a rational strategy to treat schistosomiasis. Actually, the effectiveness of combination therapy has been verified using PZQ and artemisinin derivatives, but concerns remain that the use of artemisinin derivatives may favor the emergence of multidrug-resistant malaria [39]. However, the results from this study avoid that concern since these compounds have not been used as anti-malarial drugs. Moreover, the effect of PZQ against schistosomes is reversible, which means that when the drug is removed, the worms' activity and morphology is partially restored [30]. In contrast, the effects of OAR22 and OAR27 are irreversible (data not shown). Additionally, these compounds are “lead” compounds, which only provide structural models for further development of more potent derivatives. Antischistosomal capacity could be improved through structural optimization. There have been several successful examples of improving drug activity through structural optimization in previous studies [40], [41].

In the target validation experiment, we found OAR27 could significantly inhibit r*Sj*OAR activity with a specific IC_50_ value (21.79±1.33) µg/ml, which shows a high correlation with the antischistosomal activity test as well as the BIAcore analysis. In contrast, although OAR22 showed effective antischistosomal activity, its inhibition r*Sj*OAR enzyme activity was poor. One possible explanation for this phenomenon is that *Sj*OAR is not the only drug target of OAR22. However, it was unexpected to find that OAR19 displayed no inhibition on the activity of r*Sj*OAR, which suggests that OAR19 might not target *Sj*OAR. The previously demonstrated binding capacity of OAR19 with r*Sj*OAR protein was non-specific, so the catalytic activity may not have been inhibited by binding. In this study, we also tested enzyme inhibition for all 27 small molecule compounds and tried to find whether there was a positive correlation between enzyme inhibition and parasite growth inhibition. However, we didn't observe the correlations among all the tested compounds as expected. The reason may be that many other factors such as compound molecular weight, solubility or metabolism *in vivo* could affect the results of the antischistosomal activity test.

Although the compound concentration required to kill worms was relatively high, there is still enough tolerance space compared with human cell cytotoxicity. Compounds OAR 22 and 27 exhibit almost no significant cytotoxicity at their corresponding median lethal dose (approximately 20 µg/ml). Even if the concentration were doubled from the median lethal dose (approximately 40 µg/ml), which would kill 75% to of 100% adult worms 72 hours, OAR22 and 27 only exhibit 17% and 36% cytotoxicity against human cells (Figure 8). The cytotoxicity of OAR22 and OAR27 seems strange, since OAR homologs are absent in human. However, these compounds might interact with other human proteins.

In his study, we also attempted to verify whether *Sj*OAR was a potential target through RNA interference. We designed two pairs of interference sequences and separately transformed them into juvenile worms (14-day-old) using the soaking method (incubate worms in culture medium containing 200 nM siRNA for 5 days). Although *Sj*OAR mRNA level was successfully reduced by 49.4% by one of the sequence (average value of three independent experiments), we did not observe any significant morphological alterations or worm death in the treated group compared with the control group. More interference conditions should be explored with the goal of obtaining a more complete knockdown. We changed our strategy and attempted to use chemical inhibitors to determine if *Sj*OAR was a reliable target. Hexachlorophene was reported to be a potent inhibitor of the *P. falciparum* OAR protein [22]. If we could establish a positive correlation between *Sj*OAR enzymatic inhibition and antischistosomal ability, it would also support *Sj*OAR as a potential drug target. However, further investigation revealed that hexachlorophene is not a specific inhibitor of OAR protein; it can also inhibit the enoyl-acyl carrier protein Reductase (FabI), another key enzyme in the FAS II pathway [42]. In fact, because of the structural similarity of the enzymes involved in the FAS II pathway, finding an OAR-specific inhibitor is difficult.

In summary, in this study, we characterized the *Sj*OAR protein, introduced a combined strategy to develop novel antischistosomal drugs, and identified two compounds that could be used as starting candidates for further drug development. Future studies could focus on *in vivo* treatment, design of lead compound derivatives and optimal crystallization conditions for OAR.

## Supporting Information

Figure S1 **The putative pathway for fatty acid biosynthesis in** ***S. japonicum*** **.** The star indicates the target protein investigated in this study.
(TIF)

Figure S2 **Multiple sequence alignment of** ***Sj*** **OAR with OAR from other species.** *S. japonicum* (AAW26955.1); *S. mansoni* (XP_002575617.1); *E. coli* (NP_415611.1); *M. tuberculosis* (NP_215999.1) and *P. falciparum* (XP_001352100.1). Identical amino acid sequences are highlighted in black, while similar residues are shown in gray (with 80% sequence identity). The predicted active sites are marked with asterisk (Ser150, Tyr163 and Lys167).
(TIF)

Figure S3 **Predicted structure and docking area of** ***Sj*** **OAR protein.** **A.** The structural illustration of *Sj*OAR with α-helices (red), β-sheets (yellow) and loops (green). **B.** The C-alpha backbone of the original model of SDR levodione reductase from *C. aquaticum* M-13 (PDB ID: 1IY8), shown as a tube overlayed with the *Sj*OAR model shown as a blue line trace. The conserved nature of the Rossmann fold was demonstrated by the successful docking of NAD in the *Sj*OAR active site. The figure was generated with Sybyl 8.0. NAD: nicotinamide adenine dinucleotide; MRD: 2-methyl-2, 4-pentanediol.
(TIF)

Figure S4 **Comparison of the binding of identified compounds and r** ***Sj*** **OAR using BIAcore analysis.** Compounds were dissolved at a concentration of 10 µM in HBS buffer containing 1‰ DMSO. The blank group (buffer only) and the PZQ control group were used to confirm the system's integrity. The recombinant His-tag fusion *Sj*OAR protein was immobilized onto a nitrilotriacetic acid NTA sensor chip. The relative binding capacity is presented as RelResp.
(TIF)

Figure S5 **The structures of compounds 19, 22, 27 and PZQ.**
(TIF)

Figure S6 **Time-dependent survival rates of juvenile worms treated with compounds 22, 27 and PZQ.** **A** and **B** juvenile worms were treated with compounds OAR22 and OAR27. Compound concentrations were 10 µg/ml (asterisk), 20 µg/ml (triangle), 40 µg/ml (square) and 80 µg/ml (diamond). 40 µg/ml of PZQ (dash line with circle mark) was used as a positive control.
(TIF)

Figure S7 **Optical microscopy of juvenile worms exposed to compounds 22, 27 and PZQ.** **A.** The morphology of juvenile worms treated with 2% DMSO for 72 h. **B.** The morphology of juvenile worms treated with 40 µg/ml of OAR22 for 72 h. **C.** The morphology of juvenile worms treated with 40 µg/ml of OAR27 for 48 h. **D.** The morphology of juvenile worms treated with 40 µg/ml of PZQ for 72 h.
(TIF)

Table S1 **Kinetic parameters of some of the compounds.** K_A_ is the association constant and K_D_ is the dissociation constant. R_max_ is the maximum binding capacity. Chi^2^ was used to access the fitness of experimental data, and acceptable statistics were defined as Chi^2^ less than 10% R_max_.
(DOC)
