# Supplementary figures and images for: 3-Oxoacyl-ACP Reductase from Schistosoma japonicum: Integrated In Silico-In Vitro Strategy for Discovering Antischistosomal Lead Compounds
Source: PLoS One. 2013 Jun 7;8(6):e64984. doi: 10.1371/journal.pone.0064984 (PMC3676400; doi:10.1371/journal.pone.0064984)

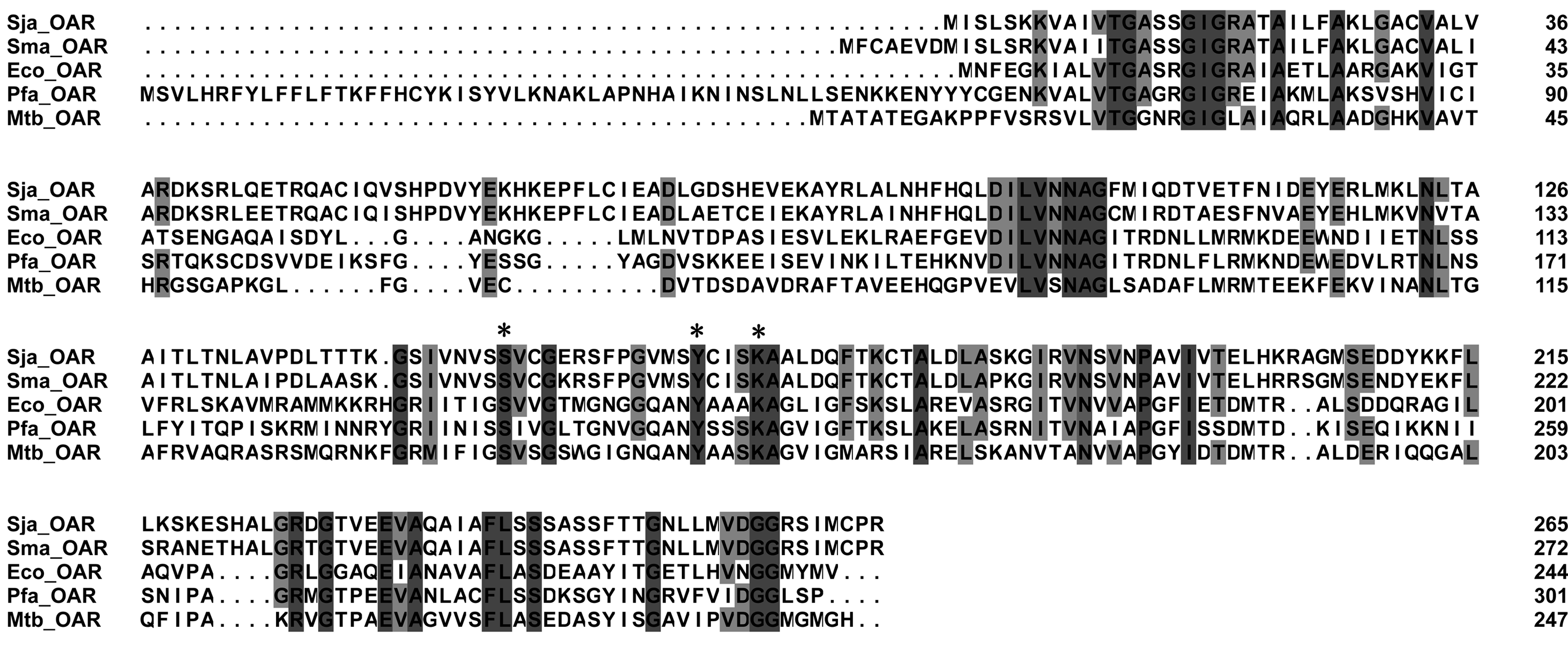

Supplement: Figure S2 — Multiple sequence alignment of Sj OAR with OAR from other species. S. japonicum (AAW26955.1); S. mansoni (XP_002575617.1); E. coli (NP_415611.1); M. tuberculosis (NP_215999.1) and P. falciparum (XP_001352100.1). Identical amino acid sequences are highlighted in black, while similar residues are shown in gray (with 80% sequence identity). The predicted active sites are marked with asterisk (Ser150, Tyr163 and Lys167). (TIF) [file pone.0064984.s002.tif]

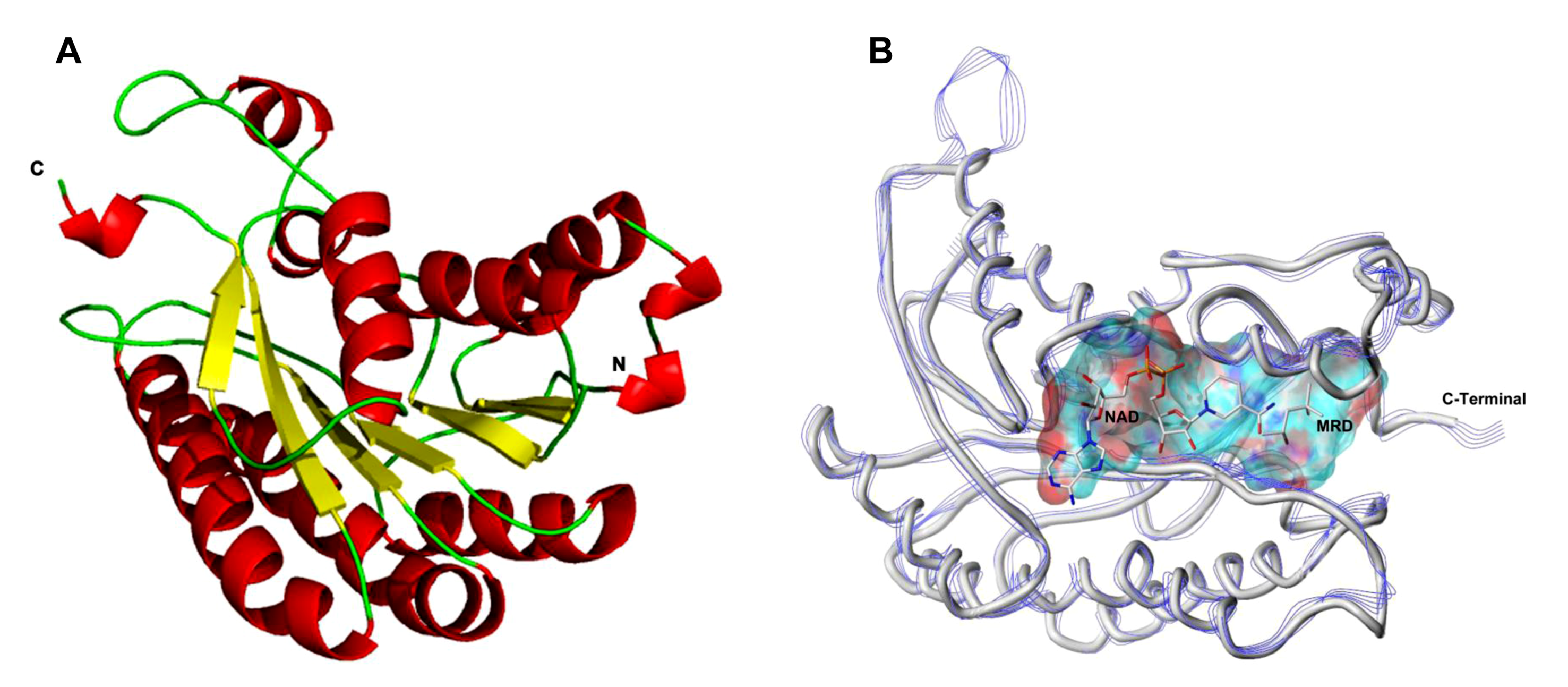

Supplement: Figure S3 — Predicted structure and docking area of Sj OAR protein. A. The structural illustration of SjOAR with α-helices (red), β-sheets (yellow) and loops (green). B. The C-alpha backbone of the original model of SDR levodione reductase from C. aquaticum M-13 (PDB ID: 1IY8), shown as a tube overlayed with the SjOAR model shown as a blue line trace. The conserved nature of the Rossmann fold was demonstrated by the successful docking of NAD in the SjOAR active site. The figure was generated with Sybyl 8.0. NAD: nicotinamide adenine dinucleotide; MRD: 2-methyl-2, 4-pentanediol. (TIF) [file pone.0064984.s003.tif]

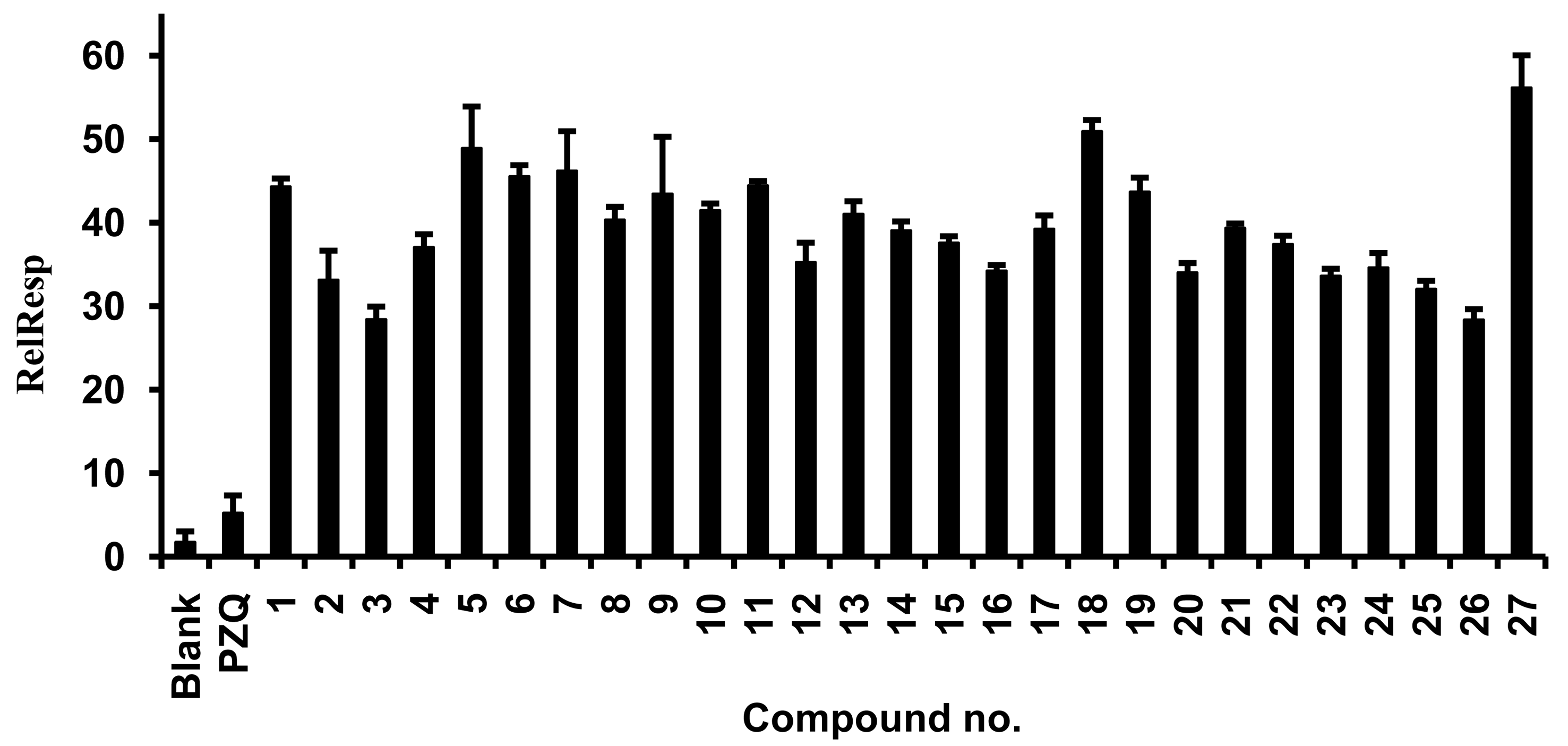

Supplement: Figure S4 — Comparison of the binding of identified compounds and r Sj OAR using BIAcore analysis. Compounds were dissolved at a concentration of 10 µM in HBS buffer containing 1‰ DMSO. The blank group (buffer only) and the PZQ control group were used to confirm the system's integrity. The recombinant His-tag fusion SjOAR protein was immobilized onto a nitrilotriacetic acid NTA sensor chip. The relative binding capacity is presented as RelResp. (TIF) [file pone.0064984.s004.tif]

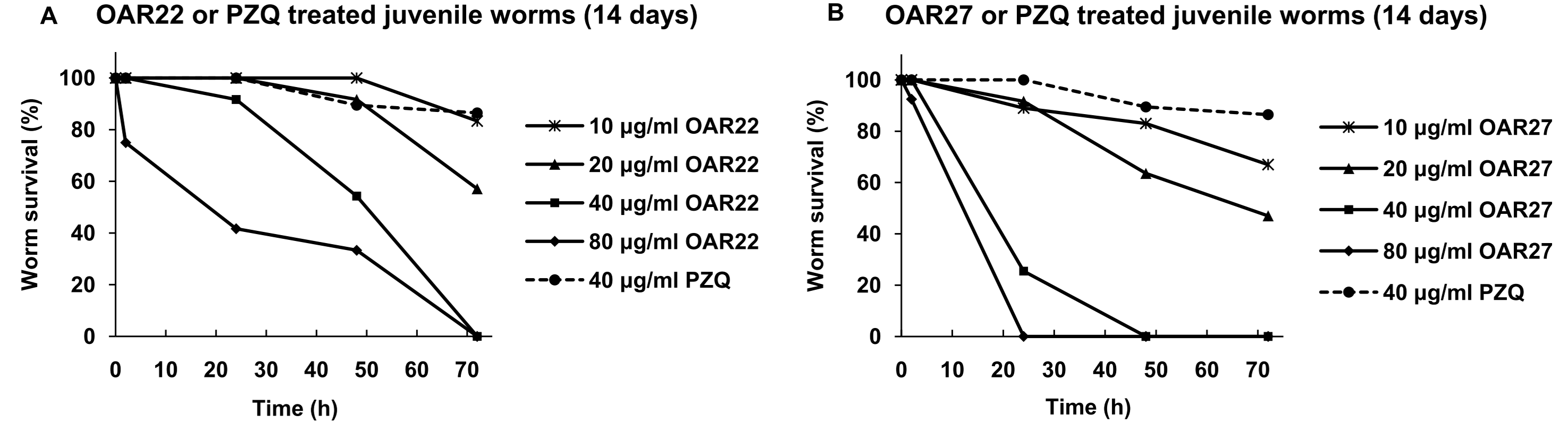

Supplement: Figure S6 — Time-dependent survival rates of juvenile worms treated with compounds 22, 27 and PZQ. A and B juvenile worms were treated with compounds OAR22 and OAR27. Compound concentrations were 10 µg/ml (asterisk), 20 µg/ml (triangle), 40 µg/ml (square) and 80 µg/ml (diamond). 40 µg/ml of PZQ (dash line with circle mark) was used as a positive control. (TIF) [file pone.0064984.s006.tif]

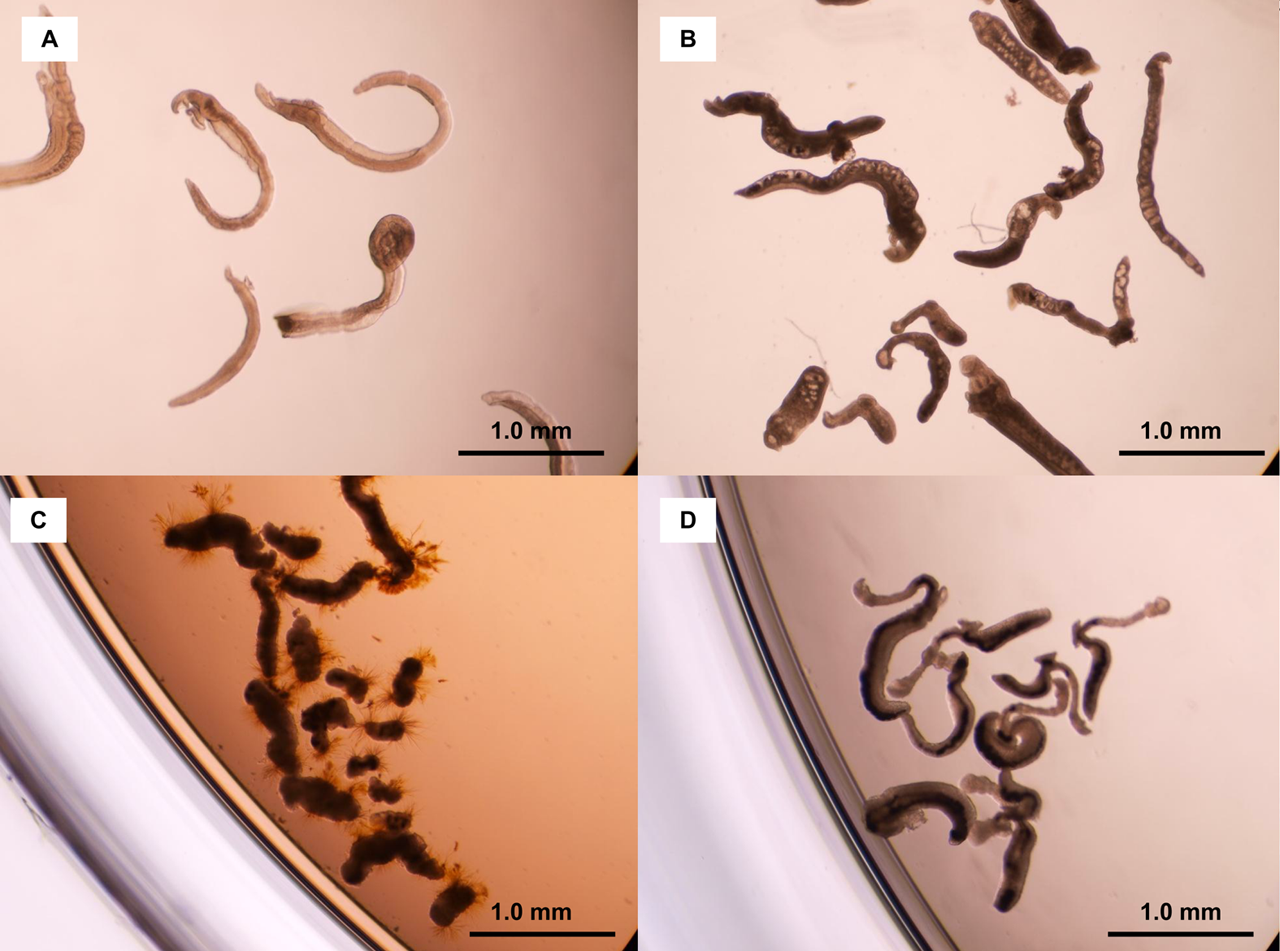

Supplement: Figure S7 — Optical microscopy of juvenile worms exposed to compounds 22, 27 and PZQ. A. The morphology of juvenile worms treated with 2% DMSO for 72 h. B. The morphology of juvenile worms treated with 40 µg/ml of OAR22 for 72 h. C. The morphology of juvenile worms treated with 40 µg/ml of OAR27 for 48 h. D. The morphology of juvenile worms treated with 40 µg/ml of PZQ for 72 h. (TIF) [file pone.0064984.s007.tif]
