# Supplementary material for: 3-Oxoacyl-ACP Reductase from Schistosoma japonicum: Integrated In Silico-In Vitro Strategy for Discovering Antischistosomal Lead Compounds
Source: PLoS One. 2013 Jun 7;8(6):e64984. doi: 10.1371/journal.pone.0064984 (PMC3676400; doi:10.1371/journal.pone.0064984)
Supplement: Table S1 — Kinetic parameters of some of the compounds. KA is the association constant and KD is the dissociation constant. Rmax is the maximum binding capacity. Chi2 was used to access the fitness of experimental data, and acceptable statistics were defined as Chi2 less than 10% Rmax. (DOC) [file pone.0064984.s008.doc]

| Compound | K_A_ (1/M) | K_D_ (M) | R_max_ | Chi^2^ |
| --- | --- | --- | --- | --- |
| OAR1 | 1.27×10^5^ | 7.90×10^-6^ | 33.1 | 5.00 |
| OAR5 | 1.89×10^5^ | 5.30×10^-6^ | 34.7 | 2.05 |
| OAR7 | 1.97×10^4^ | 5.09×10^-5^ | 113.0 | 1.16 |
| OAR8 | 8.29×10^4^ | 1.21×10^-5^ | 57.4 | 1.94 |
| OAR9 | 5.81×10^4^ | 1.72×10^-5^ | 67.6 | 3.65 |
| OAR11 | 7.15×10^4^ | 1.40×10^-5^ | 61.9 | 2.98 |
| OAR12 | 7.95×10^4^ | 1.26×10^-5^ | 57.0 | 1.74 |
| OAR13 | 6.20×10^4^ | 1.61×10^-5^ | 50.9 | 2.34 |
| OAR14 | 4.61×10^4^ | 2.75×10^-5^ | 60.2 | 0.98 |
| OAR16 | 9.03×10^4^ | 1.11×10^-5^ | 51.3 | 1.46 |
| OAR18 | 1.23×10^5^ | 8.11×10^-6^ | 44.3 | 0.29 |
| OAR19 | 1.24×10^5^ | 8.07×10^-6^ | 50.3 | 1.31 |
| OAR22 | 2.73×10^4^ | 3.66×10^-5^ | 131.0 | 0.58 |
| OAR27 | 5.41×10^7^ | 1.85×10^-8^ | 40.7 | 0.86 |
